# Chordoma Characterization of Significant Changes of the DNA Methylation Pattern

**DOI:** 10.1371/journal.pone.0056609

**Published:** 2013-03-22

**Authors:** Beate Rinner, Andreas Weinhaeusel, Birgit Lohberger, Elke Verena Froehlich, Walter Pulverer, Carina Fischer, Katharina Meditz, Susanne Scheipl, Slave Trajanoski, Christian Guelly, Andreas Leithner, Bernadette Liegl

**Affiliations:** 1 Center for Medical Research, Medical University Graz, Graz, Austria; 2 AIT – Austrian Institute of Technology, Health & Environment Department, Molecular Diagnostics Unit, Vienna, Austria; 3 Department of Orthopaedic Surgery, Medical University Graz, Graz, Austria; 4 Institute of Pathology, Medical University Graz, Graz, Austria; Shanghai Jiao Tong University School of Medicine, China

## Abstract

Chordomas are rare mesenchymal tumors occurring exclusively in the midline from clivus to sacrum. Early tumor detection is extremely important as these tumors are resistant to chemotherapy and irradiation. Despite continuous research efforts surgical excision remains the main treatment option. Because of the often challenging anatomic location early detection is important to enable complete tumor resection and to reduce the high incidence of local recurrences. The aim of this study was to explore whether DNA methylation, a well known epigenetic marker, may play a role in chordoma development and if hypermethylation of specific CpG islands may serve as potential biomarkers correlated with SNP analyses in chordoma. The study was performed on tumor samples from ten chordoma patients. We found significant genomic instability by Affymetrix 6.0. It was interesting to see that all chordomas showed a loss of 3q26.32 (PIK 3CA) and 3q27.3 (BCL6) thus underlining the potential importance of the PI3K pathway in chordoma development. By using the AITCpG360 methylation assay we elucidated 20 genes which were hyper/hypomethylated compared to normal blood. The most promising candidates were nine hyper/hypomethylated genes C3, XIST, TACSTD2, FMR1, HIC1, RARB, DLEC1, KL, and RASSF1. In summary, we have shown that chordomas are characterized by a significant genomic instability and furthermore we demonstrated a characteristic DNA methylation pattern. These findings add new insights into chordoma development, diagnosis and potential new treatment options.

## Introduction

Chordomas are malignant tumors with a phenotype that recapitulates the notochord. These tumors arise within the bones of the axial skeleton and show a destructive growth [1], [2]. Chordomas are typically largely resistant to conventional chemo- and radiotherapy and therefore surgery remains the main treatment option. However, the critical anatomic location and the commonly large tumor size rarely allow a wide curative excision. Therefore recurrent disease is a common event and even metastases have been reported in up to 40% of cases [3]. The molecular and genetic events involved in the development and progression of chordomas are not well understood and biomarkers do not exist. Although chordomas harbor common chromosomal gains and losses [4] they lack balanced or unbalanced chromosomal exchanges. Those lead to the creation of fusion genes and also screening for mutations in brachyury (a nuclear transcription factor highly expressed in chordomas) and other common cancer associated genes like KRAS and BRAF which failed to show a consistent genetic profile. DNA methylation is a tightly regulated process during normal development and it becomes deregulated during neoplastic transformation and disease development [5]. DNA methylation is relatively stable in body fluids like serum or plasma and can therefore be easily detected by sensitive PCR-based assay [6]. Hypomethylation and/or hypermethylation of specific gene loci, including tumor suppressor genes are strongly associated with disease development [7]. DNA methylation of cytosine at CpG islands can function as transcription repressor, which subsequently leads to the silencing of the associated genes.

To the best of our knowledge epigenetic data on chordomas are not available. Therefore we decided to explore if DNA methylation, a well known epigenetic marker, may play a role in chordoma development and if hypermethylation of specific CpG islands may serve as potential biomarkers correlated with single nucleotide polymorphisms (SNP) analyses in chordoma.

## Materials and Methods

### Patient samples

The Caucasian study-group included ten chordoma specimens obtained from four male and six female patients. The age of patients at time of diagnosis was between 25 to 75 years (average age 59.7). Tumors were located in the skull, the sacrum/coccyx and the mobile spine. Tumor-volume ranged from 1.5 to 668.2 cm^3^ (average 146). All ten chordomas were morphological and histological classified as classic chordomas. The follow-up period ranged from 1 to 113 months (average 41.9). All patients included in the present study were treated by surgery. Seven patients had an intralesional resection, two patients a wide, and one patient a marginal resection. Three out of ten patients received an irradiation-therapy. During the follow-up half of the patients developed a chordoma recurrence. Two patients showed lung metastases. At the end of the follow-up period four patients were DOD (death of disease), one patient suffered a DOC (death of other cause), three patients were AWD (alive with disease), and two patients had NED (no evidence of disease). The research is an original one, presently not under consideration for publication elsewhere, free of conflict of interest and conducted by the highest principles of human subjects. The study protocol and the consent of the informed patients were approved by the ethics committee of the Medical University Graz (vote #18-192ex06/07; valid until 17.04.2013). No research outside Austria was conducted. All patients were informed in detail and have given their written approval.

### Affymetrix SNP 6.0 array processing and analysis

Genomic DNA was isolated from chordoma tumor tissue and primary peripheral blood cells using the QIAmp DNA Kit (Qiagen, Hilden, Germany). Affymetrix GeneChip Human Mapping SNP 6.0 arrays were performed as described in the Genome-Wide Human SNP Nsp/Sty 6.0 User Guide (Affymetrix Inc., Santa Clara, CA). SNP 6.0 data were imported and normalized using the Genotyping Console 4.0 program default settings. All samples passing QC criteria were subsequently genotyped using the Birdseed (v2) algorithm. We used 60 raw HapMap data generated with the Affymetrix Genome-Wide Human SNP Array 6.0 as reference. Data were obtained from Affymetrix web site and used for normalization. For visualization of Copy Number state and LOH Chromosome Analysis Suite 1.1 software was used.

### DNA methylation analyses

The digestion of 600 ng genomic DNA with methylation-sensitive restriction enzymes (MSRE) was performed overnight at 37°C by employing a mixture of 6 units of each AciI (New England Biolabs, Frankfurt, Germany), Hin6I (Fermentas, St. Leon-Rot, Germany) and HpaII (Fermentas). Completion of digestion was confirmed by using a control PCR covering known differentially methylated and cancer gene regions (DMRs; H19, IGF2, ABL1, PITX2, XIST and FMR1) as published [8]. Then restriction enzymes were heat inactivated at 65°C for 20 min and digested DNA was amplified in 16 multiplex reactions covering a total of 360 5′UTR targets using biotinylated reverse primers. Amplicons of the 16 multiplex PCRs were pooled and upon agarose-gel-control mixed with hybridization buffer and hybridized onto the AIT-CpG360 microarray, presenting triplicate spots of amplicon-specific DNA probes. Upon hybridization and stringency washings, the hybridized amplicons were detected via streptavidin-Cy3 fluorescence. Microarrays were scanned and intensity data extracted from images using Genepix6.0 software (AXON). Then data were subjected to statistical analysis using BRB-AT (see section “data analysis”). Detailed information on AIT-CpG360 design and analyses is available as supplemental info (Suppl. S1); DNA sequences of primers and probes are published [9].

### High throughput quantitative PCR analysis for confirming DNA methylation changes

qPCR was performed on MSRE-digested DNA for confirmation of AIT-CpG360 microarray analyses in a nanoliter microfluidics device (running 48 qPCR assays of 48 DNA samples in parallel) using the BioMark system (Fluidigm Corporation, San Francisco, CA). qPCR confirmation was conducted upon pre-amplification of methylation sensitive restriction enzyme digested DNA using a pool of 48 primer pairs. Pre-amplification products were subjected to single gene-specific qPCRs in a BioMark Instrument using the 48.48 nanoliter qPCR devices (Fluidigm Corporation, CA) as outlined in “Methods S1”. The qPCR ct values were extracted with Real-Time PCR Analysis Software of the BioMark instrument (Fluidigm Corporation). Transformed “45-Ct” values were used for data analyses.

### Data analysis

Statistical analysis of microarray and qPCR experiments was performed using the BRB-ArrayTools software 3.8.1 developed by Dr. Richard Simon and the BRB-ArrayTools Development Team (http://linus.nci.nih.gov/brb). Values of AIT-360-CpG-arrays were log_2_-transformed and a global normalization was used to median center the log intensity values within one experiment. To identify genes, differentially methylated between patient-sample classes, a random-variance t-test for paired samples was applied to both data sets [10] [11]. Genes were considered statistically significant, if the parametric p-value was less than 0.01. Significance of differentially methylated genes was ranked using the p-value of the univariate test. In addition the false discovery rate (FDR) was calculated using the method of Benjamini and Hochberg as provided within BRB-ArrayTools software. For defining classifiers with potentially diagnostic value, “class prediction” analyses were conducted in BRB and classifiers defined by leaving one out cross validation (see also the BRB website: http://linus.nci.nih.gov/~brb/TechReport.htm).

## Results

### Chromosomal copy number variation (CNV) analysis using Affymetrix 6.0CNV/SNP arrays

**Figure 1:**
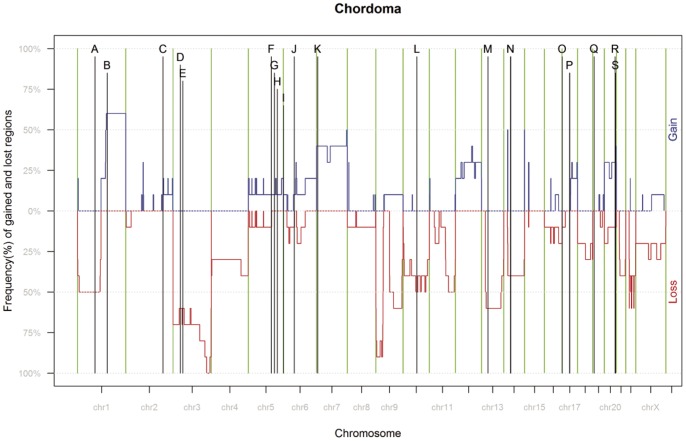
Frequency plot by genomic position. Graphical summary of chromosomal alterations (CNV and LOH) observed for the ten chordoma samples. Chromosome Y was not shown in the plot. Black line represent hyper/hypomethylated genes, whereas the letters A- S can be found in Table 3.

**Figure 2:**
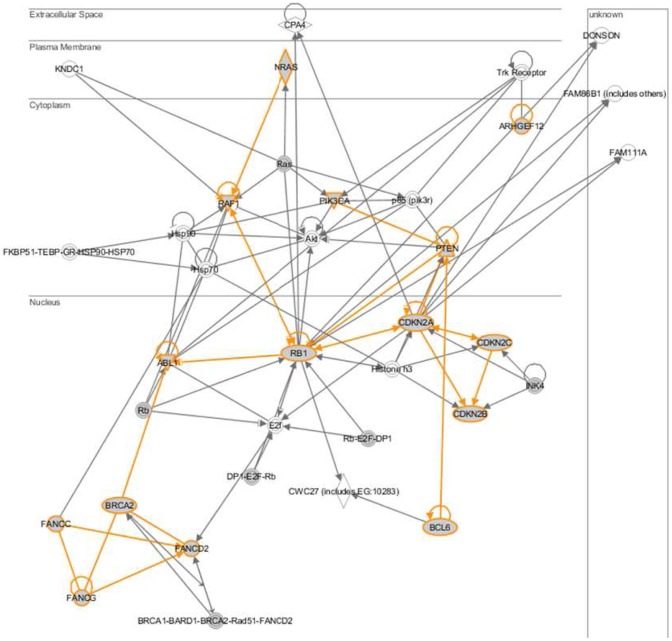
Relationship of interesting genes using IPA (Ingenuity Pathway Analysis).

Ten chordoma samples were tested for copy number (CN) and LOH using Affymetrix 6.0 CNV/SNP Arrays. Most common (>50% of the samples) chromosomal CN gains were observed for 1q21.1-q44, 7q36.3, 14q32.33, and 22q11.22 and losses for 3p26.3-q29, 9, 13q12.11-q22.1, and 22q12-q13.2. The most common chromosome loss involved chromosome 3 where 6 of 10 patients showed a loss of 3p25.2 (RAF1) and all 10 patient showed a loss of 3q26.32 (PIK 3CA) and 3q27.3 (BCL6). Table 1 summarizes the most common CN in ten chordoma patients. The cross-linking of interesting genes is shown in Figure 1 using IPA (Ingenuity Pathway Analysis) software. Furthermore, copy numbers were matched with methylation data and presented in Figure 2 to see whether a chromosome is particularly affected by CN-variation or hyper/hypo methylation pattern.

**Table 1:** Selected copy number gains/losses of ≥50 frequency. Size is expressed in megabases.

Cytogenetic Locus	Size	Gain/Loss	Associated Cancer Genes
1p36.23-p13.1	107,4903	loss	*MAD2L2, SDHB, MYCL1, MPL, PLK3,*
			*MUTYH, CDKN2C, BCL10, NRAS, NGF*
1q21.1-q44	103,65986	gain	*PRCC, NTRK1, SDHC, FH*
3p26.3-q29	197,83567	loss	*FANCD2, VHL, RAF1, XPC, TGFBR2,*
			*MLH1, CTNNB1, MITF, GATA2,*
			*AC128683.3, PIK3CA, BCL6*
7q36.2-q36.2	2,792315	gain	
9p24.3-p13.2	37,776911	loss	*JAK2, CDKN2A, CDKN2B,*
			*FANCG, PAX5*
9q21.11-q34.13	64,138573	loss	*GNAQ, FANCC, PTCH1, XPA,*
			*TGFBR1, ABL1*
10q21.3-q22.2	11,308422	loss	
10q23.2-q23.33	6,239005	loss	*BMPR1A, PTEN, FAS*
10q25.2-q25.3	4,445234	loss	
11q22.1-q24.3	29,867835	loss	*BIRC3, ATM, SDHD, MLL, ARHGEF12*
13q12.11-q33.1	84,422685	loss	*FLT3, FLT1, BRCA2, RB1,ERCC5*
14q11.2	0,633018	gain	
14q32.33	0,536743	gain	
22q11.1-q11.21	0,398458	loss	*SMARCB1, CHEK2, EWSR1,*
			*NF2, PDGFB, EP300*
22q11.23	0,100303	loss	
22q12.1-q12.3	3,359421	loss	*CHEK2, EWSR1, NF2*
22q13.1-q13.2	2,680197	loss	*EP300*

### Identification of DNA methylation changes in chordoma

We analysed 36 DNA samples and 3 negative controls using the AITCpG360 methylation assay. The aim was to identify biomarkers for serum-based patient testing. Therefore we also included healthy blood samples from volunteers in our analyses. For the identification of genes differentially methylated in chordoma versus normal blood we used “class comparison” using a cut off value on the single gene level of p<0.01 elucidated 20 genes. Four of them showed p-values below 0.001 (HIC1, CTCFL, ACTB, RASSF1). Based on the geometric mean of the chip intensities from the class of blood samples and chordoma samples the fold change between classes ranged from 0.024–3.82. Values below zero indicate hypermethylation in chordoma versus peripheral blood (inverted values range from 41.66 to 0.026 fold increase in intensities in chordoma (Table 2). It is of utmost interest for serum-cfDNA methylation based diagnostic testing of clinically suspected patients suffering from chordoma to elucidate a classifier for proper distinction between the methylation pattern of chordoma and blood-DNA to avoid false positives due to the background blood-DNA which is very likely to be the most abundant DNA population present in cell free serum. For identification and building a classifier for “prediction” of novel samples we performed “class prediction”. A feature selection was set to include only genes significantly different between the classes at p<0.01 significance level, and the “Leave-one-out cross-validation” method was used to compute misclassification rate. 94% of samples were correctly classified (sensitivity  = 100.0% and specificity  = 88.9%; AUC = 0.94) by the gene methylation classifier derived from the “diagonal linear discriminant analysis” and also from the “1-nearest neighbor” classifier. Other prediction methods (compound covariate predictor, 3-nearest neighbor, nearest centroid, support vector machines and Bayesian compound covariate predictor) made 89% correct classification possible. The classifier genes including summary statistics are listed in Table 3.

**Table 2:** Class comparison results for elucidation of differentially methylated genes in chordoma versus peripheral blood.

#	Parametric p-value	FDR	mean intensities of blood	mean intensities of chordoma	Fold-change	Gene symbol
1	1.9e-06	0.000692	117.83	5002.77	0.024	*HIC1*
2	7.87e-05	0.0143	122.83	389.47	0.32	*CTCFL*
3	0.0002284	0.024	1680.69	45724.96	0.037	*HIC1*
4	0.0002639	0.024	204.18	2114.22	0.097	*ACTB*
5	0.0005252	0.0382	99.87	2091.96	0.048	*RASSF1*
6	0.0020097	0.122	240.07	3056.36	0.079	*CDX1*
7	0.002575	0.134	298.76	122.03	2.45	*JUP*
8	0.0034824	0.148	1786.2	6777.17	0.26	*GBP2*
9	0.0038254	0.148	577.96	182.72	3.16	*NEUROG1*
10	0.0043484	0.148	9598.38	22361.92	0.43	*IRF4*
11	0.0044802	0.148	274.47	114.11	2.41	*STAT1*
12	0.0055942	0.156	69.16	181.48	0.38	*DLEC1*
13	0.0057031	0.156	132.37	592.55	0.22	*COL21A1*
14	0.0063306	0.156	3185.91	5503.58	0.58	*GNAS*
15	0.0065378	0.156	255.63	4661.49	0.055	*KL*
16	0.006866	0.156	1157.46	2159.2	0.54	*C3*
17	0.0084843	0.167	186.9	3110.51	0.06	*SRGN*
18	0.0085768	0.167	3744.86	979.1	3.82	*BAZ1A*
19	0.0096666	0.167	98.26	62.79	1.56	*HSD17B4*
20	0.0097382	0.167	3585.36	33560.67	0.11	*S100A9*

Genes significantly (p<0.01) different between classes are depicted, including the parametric p value, false discovery rate (FDR), geometric mean of class-intensities and fold changes are listed.

**Table 3:** Composition of the classifier derived from class prediction (Sorted by t -value): HIC1 presented by two different probes on the CpG360 array is present twice in two lines.

		Parametric p-value	t-value	% CV support	Geom mean of intensities in blood	Geom mean of intensities in chordoma	Fold-change	Gene symbol
1	O	1.9e-06	−7.254	100	117.83	5002.77	0.024	*HIC1*
2	R	7.87e-05	−5.254	100	122.83	389.47	0.32	*CTCFL*
3	O	0.0002284	−4.726	100	1680.69	45724.96	0.037	*HIC1*
4	K	0.0002639	−4.655	100	204.18	2114.22	0.097	*ACTB*
5	E	0.0005252	−4.323	100	99.87	2091.96	0.048	*RASSF1*
6	H	0.0020097	−3.684	100	240.07	3056.36	0.079	*CDX1*
7	A	0.0034824	−3.424	100	1786.2	6777.17	0.26	*GBP2*
8	I	0.0043484	−3.318	100	9598.38	22361.92	0.43	*IRF4*
9	D	0.0055942	−3.199	72	69.16	181.48	0.38	*DLEC1*
10	J	0.0057031	−3.189	56	132.37	592.55	0.22	*COL21A1*
11	S	0.0063306	−3.14	56	3185.91	5503.58	0.58	*GNAS*
12	M	0.0065378	−3.124	33	255.63	4661.49	0.055	*KL*
13	Q	0.006866	−3.101	50	1157.46	2159.2	0.54	*C3*
14	L	0.0084843	−3	33	186.9	3110.51	0.06	*SRGN*
15	B	0.0097382	−2.934	28	3585.36	33560.67	0.11	*S100A9*
16	F	0.0096666	2.937	28	98.26	62.79	1.56	*HSD17B4*
17	N	0.0085768	2.995	22	3744.86	979.1	3.82	*BAZ1A*
18	C	0.0044802	3.304	100	274.47	114.11	2.41	*STAT1*
19	G	0.0038254	3.379	100	577.96	182.72	3.16	*NEUROG1*
20	P	0.002575	3.567	100	298.76	122.03	2.45	*JUP*

The letters (A–S) can be found in Figure 2, where SNP data are combined with methylation data. The column “%CV-support” in the table indicates the percentage of the cross-validation training sets in which each gene was selected during “leave-one out cross validation”. 100% means that the gene is so strong that it was selected in all of the cross-validated training sets. “Geom. mean of intensities” is derived from chip intensity-values.

### qPCR confirmation of DNA methylation changes in chordoma

#### Analytical qualification of MSRE-coupled qPCR

To reconfirm the microarray-hybridization based analyses we subjected both the undigested and MSRE-digested DNA samples to qPCR analyses using nanoliter scaled microfluidic qPCR arrays in a Fluidigm 48.48 array for quantification of DNA methylation. PCR reactions were redesigned for covering at least 3 MSRE cut sites. On average 6 MSRE sites were present in amplicons and qPCR reactions were qualified according to MIQE guidelines (data not shown). Optimised qPCR conditions enabled parallel analyses of the 20 methylation marker candidate genes. By using the entire capacity of the 48.48 PCR array 28 genes were analysed in addition to the 20 classifier genes.

Of the 48 genes tested, 39 were significant (p<0.05) with an overall mean dCt between digested and undigested sample DNAs of 2.8−18.6 (corresponding to 7–416000 fold change) indicating proper digestion for qPCR based elucidation of methylation differences. The amplicons for H19, CDKN2A, IGF2, C3, SRGN, PIWIL4, GBP2, IRF4 showed 0.23–0.36 (in the enlisted order of genes; p = 0.057–0.260) fold differences. DNAJA4 was only minimally changed (0.75 fold), which is in line with the RRBS (reduced representation bisulphite sequencing) and 450 k Infinium data of several human cell lines presenting full methylation at this site for all except the HeLa-S3 Methyl-RRBS sequence track of the enlisted data within the UCSC genome browser (hg19, chr15:78,554,031–78,560,047). On the other hand, when comparing the mean-amplicon Ct values derived from undigested PB-DNA and chordoma DNA, almost all the genes did, as expected independently amplify from their biological origin and were within the range of 2 Ct values.

#### Comparison of “blood and chordoma” using MSRE coupled qPCR methylation analyses

Group wise comparison of blood (n = 7; four female, three male) and chordoma (n = 10; five male and five female) amplicon Ct values derived from qPCR upon MSRE digestion revealed 10 genes with higher than 2-fold changes corresponding to hypermethylation in tumors (fold change ranged from 3−2670); in addition to another 10 genes showed more than 2 fold hypermethylation in peripheral blood (a factor of 0.5−0.13 corresponds to 2–7fold; Table S1).

#### qPCR-confirmation of the “classifier derived from chip based screening.”

For confirmation of the 20 classifier genes derived from chip based screening qPCR-Ct values were used for class prediction. Using different classification algorithms, 88-94% of samples were correctly classified; one chordoma and one peripheral blood sample were frequently misclassified by the different prediction tools (Table S2).

For exemplification the performance of the Support Vector Machine Classifier enables correct classification of 94% samples at a sensitivity of 0.889 and a specificity of 1 (one chordoma sample was not correctly classified). The receiver operating characteristics (ROC) derived from the Bayesian Compound Covariate Predictor provides an area under the curve AUC of 0.952. Although the parametric p-values of several single gene qPCR ct values were below p<0.05, the classification success is very impressive.

Generation of a novel classifier from the entire set of 48 qPCR amplicons applying the feature selection criteria “Genes with univariate misclassification rate below 0.2” for class prediction elucidates a classifier of 23 genes enabling perfect classification of the entire set of study samples (AUC = 1) by the Compound Covariate Predictor, the 1-Nearest Neighbor and the Bayesian Compound Covariate Predictor. Correct classification of 94% was obtained by using the Diagonal Discriminant, the Nearest Centroid, and the Support Vector Machines analyses. The 3-Nearest Neighbor classification success was 88% (Table S3). For reducing the classifier to a lower number of genes feature selection by “univariate p-value <0.05 and 2 fold -change between classes” was applied and class prediction was performed again on the entire set of all the 48 amplicons used for qPCR. Thereby a classifier for distinction between peripheral blood and chordoma was generated. This classifer consisted of qPCR-ct methylation measures of RASSF1, KL, C3, HIC1, RARB, TACSTD2, XIST, and FMR1 (Table 4). That classifier enabled perfect classification of the set of study samples (AUC = 1) by the 1-Nearest Neighbor method. Correct classification of 94% was obtained by using the Compound Covariate Predictor and the Support Vector Machines. The classification success was 88% achieved by the Diagonal Discriminant Analyses, the Nearest Centroid, and analyses and the 3-Nearest Neighbors classifier. The Bayesian Compound Covariate Predictor allowed also perfect classification. Two samles, however, could not be classified (indicated as “NA” in Table S4).

**Table 4:** Composition of the classifier derived from class prediction (sorted by t -value): For feature selection the “univariate p-value <0.05 and 2 fold -change between classes” was applied. The column

	Parametric p-value	t-value	% CV support	Geom mean of intensities in *chordoma*	Geom mean of intensities in *blood*	Fold-change	Gene symbol
1	0.0150536	−2.77	100	1398381572.97	184247873471.87	0.0076	*C3*
2	0.0182198	−2.672	100	6375978837.27	34804904883.29	0.18	*XIST*
3	0.0330808	−2.364	50	417345518.9	12040221110.61	0.035	*TACSTD2*
4	0.0412258	2.248	44	11770399604.19	406469246.17	28.96	*FMR1*
5	0.0295713	2.422	75	5239084212.46	240494470.75	21.78	*HIC1*
6	0.0227937	2.557	94	34359738368	2005658405.4	17.13	*RARB*
7	0.0184278	2.666	100	152757376.44	703546.19	217.12	*DLEC1*
8	0.0070848	3.15	100	26322102103.28	1053729641.19	24.98	*KL*
9	5.53e-05	5.695	100	2047350879.5	100421.9	20387.49	*RASSF1*

The column “%CV-support” in the table indicates the percentage of the cross-validation training sets in which each gene was selected during “leave-one out cross validation”. 100% means that the gene is so strong that it was selected in all of the cross-validated training sets. “Geom mean of intensities” is derived from transformed qPCR-Ct values.

## Discussion

The presented study was conducted to investigate SNPs as well as hyper/hypomethylated genes in ten chordoma patients. The first part of the study focused on SNP analyses. We confirmed copy number alterations known in chordomas and describe novel gains and losses. In accordance to Le et al. who reported array comparative genomic hybridization (CGH) in 21 sporadic chordomas and found large copy number losses on chromosomes 1p, 3, 4, 9, 10, 13, 14, and 18, we were also able to demonstrate common losses including chromosome 1, 4, 9, 10, 13, 14, 18, 20, and 22 as well as common gains in 7, 12, and 19. Interestingly, in 100% of our cases chromosome 3 (3p26.3-q29) was lost. These findings are also comparable with data previously reported by Hallor et al. and Le et al. [4], [12]. Copy number gains/losses present in more than ≥50% of patients of well-known cancer associated genes for example CDKN2A, CDKN2B, RAF1 and PTEN were found and may play an important role in chordoma development. Overall, chromosome 3 shows the majority of genetic alterations. The common involvement of this chromosome has been already described in early studies by demonstrating a 3p loss [13], [14] In addition losses on chromosome 3 relating PIK3CA and BCL6 were obtained. Currently there are several inhibitors of the PI3K (phosphatidylinositol 3-kinase) pathway under investigation in solid tumours [15]. Although cross talk of the PI3K pathway with other pathways in particular the RAS/RAF/MEK pathways have been reported, inhibition of the PI3K pathway could be an attractive therapeutic target and is definitely worth further investigations. BCL6 is a transcriptional repressor binding DNA through zinc fingers and regulates transcription through interacting with other factors like Jun proteins and histone deacetylase family proteins [16], [17]. Usually BCL6 is associated with normal and abnormal B-cell development. However, Chamdin et al. showed that BCL6 arrests the differentiation of neural crest cells in neuroblastoma (NB) and may therefore play a similar role in chordoma development [18]. By merging the data, it's apparent that also RB1 (retinoblastoma) signalling plays a central role in chordoma oncogenesis [4], [12].

We were able to show that chordomas are characterized by significant genomic instability. Although a common pattern of genetic changes could be demonstrated, a consistent genetic change in all samples was not identified.

The second part of the study provides the first evidences that DNA methylation of tumor suppressor genes exit in chordomas and may serve as a marker for early tumor detection.

Early tumor detection is extremely important for chordoma patients, because these tumors are resistant to chemotherapy and irradiation. Surgical excision remains the main treatment option and based on the challenging anatomic location early detection is important to allow complete resection and to reduce the high incidence of the local recurrence. Therefore, the aim was to identify hypermethylated genes that could serve as biomarkers for early tumor detection to optimize patients' treatment. We used blood from healthy volunteers as comparison, due to the fact that notochord as comparatively tissue was not available.

DNA methylation has already provided useful biomarkers for diagnosing cancer, monitoring treatment and predicting the prognosis. Aberrant DNA hypermethylation of CpG islands in the promoter region of genes is well established as a common mechanism for the silencing of tumor suppressor genes in cancer and serve as an alternative mechanism of functional inactivation.

By comparing methylation patterns of blood from healthy individuals and chordoma patients we found 20 significantly differentially methylated genes; 15 hypermethylated in chordoma (for example RASSF1, KL, RARB, HIC1, and FMR1) and 5 hypomethylated (HSD17B4, BAZIA, STAT1, NEUROGL, and JUP).

RASSF1, KL, and HIC1 are known to be tumor suppressor genes. The inactivation of tumor suppressor genes is usually accompanied by a copy of the gene mutations and loss of the corresponding allele [19]. RASSF1 encodes a protein similar to the RAS effector proteins. In normal cells RASSF1 (Ras association domain family1 protein) a tumor suppressor gene is involved in controlling cell cycle and in repairing DNA [20]. RASSF1 has been shown to be transcriptionally silenced by promoter methylation and are frequently methylated in various tumor types. Especially in breast and colorectal cancer [21], [22], inactivation of this gene was found to be correlated with CpG-island promoter region hypermethylation.

Another tumor suppressor gene KL (klotho) is a single pass type I transmembrane protein that is localize at the plasma membrane as well as in the cytoplasm. It was initially identified as anti-senescence gene [23]. Recently, reduced KL gene expression was shown to contribute to tumorigenesis. KL has been found to function as tumor suppressor in various cancers like breast, pancreas, lung, and cervix [24]. This transmembrane protein can be shed, act as circulating hormone and is a modulator of the IGF-1 (insulin-like growth factor IGF-1) and the FGF (fibroblast growth factor) pathways. Those have recently been demonstrated to be activated in chordomas [25], [26]. KL potently inhibits ligand-dependent activation of the insulin and IGF-1 pathways [27], [28] and binds to FGFR (fibroblast growth factor receptors) [28], [29].

Another tumor suppressor gene, the HIC1 (Hypermethylated in Cancer 1) gene, is a transcriptional target of p53 and is frequently deleted or hypermethylated in various solid tumors, including colon, lung, breast, brain, and kidney [30]. We have applied this methylation assay to several other cancerous diseases [31]–[33] and could delineate several candidate-biomarker panels for the different settings. Nikolaidis et al. showed the impact of DNA methylation-based assays in the diagnosis of cytologically occult lung neoplasms [34]. HIC1 methylation could be used as a target for pharmacologic DNA-methyltransferase and could therefore suit as a potential new target to treat chordoma patients. In summary, we have shown that chordomas are characterized by significant changes of the DNA methylation pattern. A multigene DNA methylation based classifier suitable to distinguish healthy blood and chordoma DNA presented here will add a new dimension for chordoma diagnosis and treatment. We believe that our findings should be explored to circulating tumor cells or circulating cell free DNA found in peripheral blood, serum or plasma of patients, to improve chordoma diagnoses and disease monitoring. Although validation of results has to be conducted on additional patient sample cohorts and serum cfDNA, we think that the DNA methylation classifiers elucidated here, could be useful novel biomarkers advancing diagnostic workup for patients.

## Supporting Information

Table S1 **HTqPCR derived data of MSRE digested and undigested chordoma and blood DNA samples.** Mean “45-Ct” values of “classes” upon amplification are listed. The values >2 in the column “Fold Difference of chordoma digested“ versus “blood digested” indicate hypermethylation in chordomas; fold difference <0,5 indicate hypomethylation in chordomas compared to blood DNA.
(DOC)

Table S2 **Class prediction.**
(DOC)

Table S3 **Class prediction.**
(DOC)

Table S4 **Class prediction.**
(DOC)

Methods S1 **Methylation sensitive restriction enzyme digestion.**
(DOC)

## References

[1] Chugh R, Tawbi H, Lucas DR, Biermann JS, Schuetze SM, et al (2007) Chordoma: the nonsarcoma primary bone tumor. Oncologist 12: 1344–1350. 18055855 10.1634/theoncologist.12-11-1344

[2] Fletcher CD, Mertens F, Unni KK (2002) Pathology and Genetics of Tumours of Soft Tissue and Bone Vol. 5 : World Health Organization Classification of Tumours.

[3] Catton C, O'Sullivan B, Bell R, Laperriere N, Cummings B, et al (1996) Chordoma: long-term follow-up after radical photon irradiation. Radiother Oncol 41: 67–72. 8961370 10.1016/s0167-8140(96)91805-8

[4] Hallor KH, Staaf J, Jonsson G, Heidenblad M, Vult von Steyern F, et al (2008) Frequent deletion of the CDKN2A locus in chordoma: analysis of chromosomal imbalances using array comparative genomic hybridisation. Br J Cancer 98: 434–442. 18071362 10.1038/sj.bjc.6604130 PMC2361468

[5] Dhe-Paganon S, Syeda F, Park L (2011) DNA methyl transferase 1: regulatory mechanisms and implications in health and disease. Int J Biochem Mol Biol 2: 58–66. 21969122 PMC3180029

[6] Ransohoff DF (2003) Developing molecular biomarkers for cancer. Science 299: 1679–1680. 12637728 10.1126/science.1083158

[7] Herman JG, Baylin SB (2003) Gene silencing in cancer in association with promoter hypermethylation. N Engl J Med 349: 2042–2054. 14627790 10.1056/NEJMra023075

[8] Weinhaeusel A, Thiele S, Hofner M, Hiort O, Noehammer C (2008) PCR-based analysis of differentially methylated regions of GNAS enables convenient diagnostic testing of pseudohypoparathyroidism type Ib. Clin Chem 54: 1537–1545. 18617581 10.1373/clinchem.2008.104216

[9] Weinhaeusel A, Thiele S, Hofner M, Hiort O, Noehammer C (2008) PCR-based analysis of differentially methylated regions of GNAS enables convenient diagnostic testing of pseudohypoparathyroidism type Ib. Clin Chem 54: 1537–1545. 18617581 10.1373/clinchem.2008.104216

[10] Wright GW, Simon RM (2003) A random variance model for detection of differential gene expression in small microarray experiments. Bioinformatics 19: 2448–2455. 14668230 10.1093/bioinformatics/btg345

[11] Simon R, Lam A, Li MC, Ngan M, Menenzes S, et al (2007) Analysis of gene expression data using BRB-ArrayTools. Cancer Inform 3: 11–17. 19455231 PMC2675854

[12] Le LP, Nielsen GP, Rosenberg AE, Thomas D, Batten JM, et al (2011) Recurrent chromosomal copy number alterations in sporadic chordomas. PLoS One 6: e18846. 21602918 10.1371/journal.pone.0018846 PMC3094331

[13] Dalpra L, Malgara R, Miozzo M, Riva P, Volonte M, et al (1999) First cytogenetic study of a recurrent familial chordoma of the clivus. Int J Cancer 81: 24–30. 10077147 10.1002/(sici)1097-0215(19990331)81:1<24::aid-ijc5>3.0.co;2-o

[14] Scheil S, Bruderlein S, Liehr T, Starke H, Herms J, et al (2001) Genome-wide analysis of sixteen chordomas by comparative genomic hybridization and cytogenetics of the first human chordoma cell line, U-CH1. Genes Chromosomes Cancer 32: 203–211. 11579460 10.1002/gcc.1184

[15] Naumann RW (2011) The role of the phosphatidylinositol 3-kinase (PI3K) pathway in the development and treatment of uterine cancer. Gynecol Oncol 123: 411–420. 21903247 10.1016/j.ygyno.2011.08.002

[16] Chang CC, Ye BH, Chaganti RS, Dalla-Favera R (1996) BCL-6, a POZ/zinc-finger protein, is a sequence-specific transcriptional repressor. Proc Natl Acad Sci USA 93: 6947–6952. 8692924 10.1073/pnas.93.14.6947 PMC38914

[17] Vasanwala FH, Kusam S, Toney LM, Dent AL (2002) Repression of AP-1 function: a mechanism for the regulation of Blimp-1 expression and B lymphocyte differentiation by the B cell lymphoma-6 protooncogene. J Immunol 169: 1922–1929. 12165517 10.4049/jimmunol.169.4.1922

[18] Chamdin A, Jarzembowski JA, Subramanian C, Kuick R, Lee JS, et al (2009) Bcl6 is expressed in neuroblastoma: tumor cell type-specific expression predicts outcome. Transl Oncol 2: 128–137. 19701497 10.1593/tlo.08220 PMC2730138

[19] Cavenee WK (1991) Charles S. Mott Prize. Recessive mutations in the causation of human cancer. Cancer 67: 2431–2435. 2015543 10.1002/1097-0142(19910515)67:10<2431::aid-cncr2820671005>3.0.co;2-#

[20] Agathanggelou A, Cooper WN, Latif F (2005) Role of the Ras-association domain family 1 tumor suppressor gene in human cancers. Cancer Res 65: 3497–3508. 15867337 10.1158/0008-5472.CAN-04-4088

[21] Agrawal A, Murphy RF, Agrawal DK (2007) DNA methylation in breast and colorectal cancers. Mod Pathol 20: 711–721. 17464311 10.1038/modpathol.3800822

[22] Lehmann U, Langer F, Feist H, Glockner S, Hasemeier B, et al (2002) Quantitative assessment of promoter hypermethylation during breast cancer development. Am J Pathol 160: 605–612. 11839581 10.1016/S0002-9440(10)64880-8 PMC1850646

[23] Kurosu H, Kuro-o M (2008) The Klotho gene family and the endocrine fibroblast growth factors. Curr Opin Nephrol Hypertens 17: 368–372. 18660672 10.1097/MNH.0b013e3282ffd994

[24] Wang YA (2006) Klotho, the long sought-after elixir and a novel tumor suppressor? Cancer Biol Ther 5: 20–21. 16410720 10.4161/cbt.5.1.2430

[25] Sommer J, Itani DM, Homlar KC, Keedy VL, Halpern JL, et al (2010) Methylthioadenosine phosphorylase and activated insulin-like growth factor-1 receptor/insulin receptor: potential therapeutic targets in chordoma. J Pathol 220: 608–617. 20140939 10.1002/path.2679

[26] Scheipl S, Froehlich EV, Leithner A, Beham A, Quehenberger F, et al (2012) Does insulin-like growth factor 1 receptor (IGF-1R) targeting provide new treatment options for chordomas? A retrospective clinical and immunohistochemical study. Histopathology 60: 999–1003. 22372631 10.1111/j.1365-2559.2012.04186.x

[27] Wolf I, Levanon-Cohen S, Bose S, Ligumsky H, Sredni B, et al (2008) Klotho: a tumor suppressor and a modulator of the IGF-1 and FGF pathways in human breast cancer. Oncogene 27: 7094–7105. 18762812 10.1038/onc.2008.292

[28] Kurosu H, Yamamoto M, Clark JD, Pastor JV, Nandi A, et al (2005) Suppression of aging in mice by the hormone Klotho. Science 309: 1829–1833. 16123266 10.1126/science.1112766 PMC2536606

[29] Kuro-o M, Matsumura Y, Aizawa H, Kawaguchi H, Suga T, et al (1997) Mutation of the mouse klotho gene leads to a syndrome resembling ageing. Nature 390: 45–51. 9363890 10.1038/36285

[30] Wales MM, Biel MA, El Deiry W, Nelkin BD, Issa JP, et al (1995) p53 activates expression of HIC-1, a new candidate tumour suppressor gene on 17p13.3. Nat Med 1: 570–577. 7585125 10.1038/nm0695-570

[31] Weinhaeusel A, Noehammer C (2009) Methylation Assay. European Patent Application EP 09450020.4. filed 26-1-2009; WO2010086389 A1 (2010-08-05).

[32] Neumann L, Weinhäusel A, Thomas S, Horsthemke B, Lohmann DR, et al (2011) EFS shows biallelic methylation inuveal melanoma with poor prognosis as well as tissue-specific methylation. BMC Caner 11: 380–391. 10.1186/1471-2407-11-380 PMC3175225 21871071

[33] Weinhaeusel A (2010) Lung Cancer Methylation Markers. PCT-Application PCT/EP2010/051032. filed 28-1-2010. WO2010086388 A1 (2010-08-05).

[34] Nikolaidis G, Raji OY, Markopoulou S, Gosney JR, Bryan J, et al (2012) DNA methylation biomarkers offer improved diagnostic efficiency in lung cancer. Cancer Res 72(22): 5692–701 doi:10.1158/0008-5472.CAN-12-2309. Epub 2012 Sep 7 22962272 10.1158/0008-5472.CAN-12-2309 PMC3500566

