# Supplementary material for: Chordoma Characterization of Significant Changes of the DNA Methylation Pattern
Source: PLoS One. 2013 Mar 22;8(3):e56609. doi: 10.1371/journal.pone.0056609 (PMC3606365; doi:10.1371/journal.pone.0056609)
Supplement: Table S1 — HTqPCR derived data of MSRE digested and undigested chordoma and blood DNA samples. Mean “45-Ct” values of “classes” upon amplification are listed. The values >2 in the column “Fold Difference of chordoma digested“ versus “blood digested” indicate hypermethylation in chordomas; fold difference <0,5 indicate hypomethylation in chordomas compared to blood DNA. (DOC) [file pone.0056609.s001.doc]

| **Gene Row** | **Gene symbol** | **mean of log intensities for chordoma digest** | **mean of log intensities**  **for**  **chordoma**  **undigested** | **mean of log intensities for**  **blood**  **digest** | **mean of log intensities**  **for**  **blood**  **undigested** | **Fold Difference  of „chordoma digested“ vs. “blood digested”** |
| --- | --- | --- | --- | --- | --- | --- |
| 4 | [CDX1](http://www.ncbi.nlm.nih.gov/entrez/query.fcgi?db=unigene&term=CDX1) | 24.14481 | 22.38076 | 19.88451 | 23.47205 | 19.16 |
| 7 | [HIST1H2AG](http://www.ncbi.nlm.nih.gov/entrez/query.fcgi?db=unigene&term=HIST1H2AG) | 20.6608 | 26.28784 | 18.31747 | 26.21135 | 5.07 |
| 11 | [BAZ1A](http://www.ncbi.nlm.nih.gov/entrez/query.fcgi?db=unigene&term=BAZ1A) | 18.57643 | 25.83306 | 16.96642 | 25.79551 | 3.05 |
| 12 | [LAMC2](http://www.ncbi.nlm.nih.gov/entrez/query.fcgi?db=unigene&term=LAMC2) | 20.55219 | 24.53412 | 18.87455 | 24.97916 | 3.20 |
| 14 | [RASSF1](http://www.ncbi.nlm.nih.gov/entrez/query.fcgi?db=unigene&term=RASSF1) | 26.65048 | 25.03574 | 15.26645 | 25.25054 | 2672.60 |
| 18 | [ESR1](http://www.ncbi.nlm.nih.gov/entrez/query.fcgi?db=unigene&term=ESR1) | 23.20593 | 26.76519 | 20.79713 | 26.55687 | 5.31 |
| 22 | [HIC1](http://www.ncbi.nlm.nih.gov/entrez/query.fcgi?db=unigene&term=HIC1) | 23.13808 | 20.55627 | 17.30234 | 20.97878 | 57.11 |
| 33 | [S100A9](http://www.ncbi.nlm.nih.gov/entrez/query.fcgi?db=unigene&term=S100A9) | 26.77074 | 25.9937 | 24.73109 | 25.37426 | 4.11 |
| 40 | [DLEC1](http://www.ncbi.nlm.nih.gov/entrez/query.fcgi?db=unigene&term=DLEC1) | 24.09926 | 26.51209 | 19.61771 | 26.50773 | 22.34 |
| 45 | [STAT1](http://www.ncbi.nlm.nih.gov/entrez/query.fcgi?db=unigene&term=STAT1) | 18.80204 | 25.9818 | 16.22174 | 25.70422 | 5.98 |
| 5 | [TERT](http://www.ncbi.nlm.nih.gov/entrez/query.fcgi?db=unigene&term=TERT) | 22.92051 | 25.78413 | 24.19349 | 25.57056 | 0.41 |
| 6 | [CTCFL](http://www.ncbi.nlm.nih.gov/entrez/query.fcgi?db=unigene&term=CTCFL) | 18.67066 | 16.42294 | 19.84387 | 17.00463 | 0.44 |
| 13 | [ARMCX2](http://www.ncbi.nlm.nih.gov/entrez/query.fcgi?db=unigene&term=ARMCX2) | 17.498 | 19.82855 | 19.07137 | 19.8145 | 0.34 |
| 15 | [EFS](http://www.ncbi.nlm.nih.gov/entrez/query.fcgi?db=unigene&term=EFS) | 25.95855 | 25.18045 | 27.70162 | 25.1043 | 0.30 |
| 24 | [C3](http://www.ncbi.nlm.nih.gov/entrez/query.fcgi?db=unigene&term=C3) | 24.69481 | 23.84497 | 26.68261 | 20.77553 | 0.25 |
| 25 | [DCC](http://www.ncbi.nlm.nih.gov/entrez/query.fcgi?db=unigene&term=DCC) | 10.49563 | 14.2738 | 13.43665 | 15.0158 | 0.13 |
| 26 | [H19](http://www.ncbi.nlm.nih.gov/entrez/query.fcgi?db=unigene&term=H19) | 25.22713 | 23.15954 | 26.40097 | 22.86923 | 0.44 |
| 32 | [KRT17](http://www.ncbi.nlm.nih.gov/entrez/query.fcgi?db=unigene&term=KRT17) | 26.3257 | 25.35615 | 27.42754 | 25.23306 | 0.47 |
| 39 | [TP53](http://www.ncbi.nlm.nih.gov/entrez/query.fcgi?db=unigene&term=TP53) | 17.07729 | 25.29726 | 18.09062 | 25.61602 | 0.50 |
| 48 | [XIST](http://www.ncbi.nlm.nih.gov/entrez/query.fcgi?db=unigene&term=XIST) | 19.70579 | 18.17636 | 21.19647 | 17.69458 | 0.36 |

Table S1
