# Supplementary material for: Chordoma Characterization of Significant Changes of the DNA Methylation Pattern
Source: PLoS One. 2013 Mar 22;8(3):e56609. doi: 10.1371/journal.pone.0056609 (PMC3606365; doi:10.1371/journal.pone.0056609)
Supplement: Table S4 — Class prediction. (DOC) [file pone.0056609.s004.doc]

| **Class label** | **Mean numbers of genes in classifier** | **Compound Covariate Predictor** | **Diagonal Linear Discriminant Analysis** | **1-Nearest Neighbor** | **3-Nearest Neighbors** | **Nearest Centroid** | **Support Vector Machines** | **Bayesian Compound Covariate Predictor** |
| --- | --- | --- | --- | --- | --- | --- | --- | --- |
| chordoma | 7 | YES | YES | YES | YES | YES | YES | YES |
| chordoma | 6 | YES | YES | YES | YES | YES | YES | YES |
| chordoma | 10 | YES | YES | YES | YES | YES | YES | YES |
| chordoma | 14 | YES | YES | YES | NO | NO | NO | YES |
| chordoma | 10 | YES | YES | YES | YES | YES | YES | YES |
| chordoma | 9 | YES | YES | YES | YES | YES | YES | YES |
| chordoma | 10 | YES | YES | YES | YES | YES | YES | YES |
| chordoma | 8 | YES | YES | YES | YES | YES | YES | YES |
| chordoma | 8 | YES | YES | YES | YES | YES | YES | YES |
| blood | 9 | YES | NO | YES | YES | YES | YES | NA |
| blood | 8 | YES | YES | YES | YES | YES | YES | YES |
| blood | 6 | YES | YES | YES | YES | YES | YES | YES |
| blood | 8 | YES | YES | YES | YES | YES | YES | YES |
| blood | 10 | NO | NO | YES | NO | NO | YES | NA |
| blood | 9 | YES | YES | YES | YES | YES | YES | YES |
| blood | 10 | YES | YES | YES | YES | YES | YES | YES |
| **Mean percent of correct classification:** | | **94** | **88** | **100** | **88** | **88** | **94** | **100** |

Table S4
