# Supplementary material for: Chordoma Characterization of Significant Changes of the DNA Methylation Pattern
Source: PLoS One. 2013 Mar 22;8(3):e56609. doi: 10.1371/journal.pone.0056609 (PMC3606365; doi:10.1371/journal.pone.0056609)
Supplement: Methods S1 — Methylation sensitive restriction enzyme digestion. (DOC) [file pone.0056609.s005.doc]

**Supplementary data**

**SUPPLEMENTAL Methods**

**High throughput quantitative PCR analysis for confirming DNA methylation changes**

**Methylation sensitive restriction enzyme digestion:**

Of each Sample 100 ng were digested on 37°C over night in a Biometra T3000 Thermocycler (Labrepco, Horsham, PA). The reaction volume was 5 µl containing 2.5 µl DNA, 0.1 µl HpaII (10 U/µl; Fermentas, St. Leon-Rot, Germany), 0.1 µl Hin6I (10 U/µl; Fermentas), 0.1 µl AciI (10 U/µl; New England BioLabs, Ipswich, MA), 0.1 µl Hpy4IV (10 U/µl; New England BioLabs), 0.5 µl 10 x Buffer Tango (Fermentas) and 1.6 µl H_2_O. The reactions were thermally inactivated by heating them to 65°C for 20 minutes.

**Pre-Amplification:**

For the pre-amplification each forward and reverse primer pair of genes enlisted in the table were combined and diluted to a final concentration of 20 µM each.

| **List of 48 Genes used in the Experiment:** | | | | | |
| --- | --- | --- | --- | --- | --- |
| SERPINI1 | SMAD3 | DNAJA4 | DCC | S100A9 | KL |
| BOLL | NEUROG1 | ESR1 | H19 | GNAS | SRGN |
| ACTB | BAZ1A | TIMP1 | CALCA | GBP2 | IRF4 |
| CDX1 | LAMC2 | CDKN2A | FHL2 | FMR1 | RARB |
| TERT | ARMCX2 | IGF2 | PIWIL4 | SNRPN | STAT1 |
| CTCFL | RASSF1 | HIC1 | COL21A1 | TACSTD2 | PTTG1 |
| HIST1H2AG | EFS | CD24 | JUP | TP53 | HSD17B4 |
| BRCA1 | PGR | C3 | KRT17 | DLEC1 | XIST |

Equal volumes of all primer pairs were combined and diluted to a final concentration of 200 nM. The reaction volume was 25 µl containing 5 µl DNA from the previous reaction, 2.5 µl 10 x PCR Buffer without MgCl_2_ (Qiagen, Hilden, Germany), 2 µl dNTPs (2 mM; Roche, Vienna, Austria), 1.25 µl DMSO (Sigma Aldrich, Vienna, Austria), 0.15 µl Hot Star Taq (5 U/µl; Qiagen), 6.25 µl Primer Mix (200 nM) and 7.85 µl H_2_O. The PCR amplification was started with an activation step of 15 minutes at 95°C followed by 14 cycles of 95°C for 15 seconds and 65°C for 4 minutes. The pre-amplified DNA was diluted 1:5 before it was used for the Biomark.

**High throughput qPCR using the Biomark Instrument:**

For the Biomark run each forward and reverse primer pair was combined and diluted to a final concentration of 20 µM each. The “Assay Mix” was prepared in a 96 well plate. The volume of each assay was 6 µl containing of 3 µl 2 x Assay loading Reagent (Fluidigm **Corporation,** San Francisco, CA), 0.3 µl 1x DNA Suspension Buffer and 2.7 µl 20 µM of Forward and Reverse Primer Mix. For the „Sample-Mix“ all reactions were prepared in a 96 well plate. The volume of each reaction was 6 µl containing 1.5 µl of pre-amplified and diluted DNA, 0.6 µl 10x PCR Buffer containing 15 mM MgCl_2_ (Qiagen), 0.48 µl dNTPs (2 mM, Roche), 0.3 µl DMSO (Sigma Aldrich), 0.33 µl Eva Green, 20x in water (Biotium), 0.036 µl Hot Star Taq (5 U/µl; Qiagen), 0.004 µl ROX Reference Dye (Invitrogen, Vienna, Austria), 0.6 µl 20x DNA Binding Dye Sample Loading Reagent (Fluidigm **Corporation**), 2.15 µl H_2_O. 5 µl of all assays were loaded on a Biomark GE 48.48 Chip (Fluidgm **Corporation**) on the assay side and 5 µl of all sample mixes were loaded on a Biomark GE 48.48 Chip (Fluidgm **Corporation**) on the sample side. The amplification on the Biomark starts with an initial step of 2 minutes at 50°C and 10 minutes at 95°C followed by 35 cycles of 15 seconds at 95°C and 1 minute at 65°C. The melting curve was started at 65°C for 3 seconds. From there on the temperature was increased to 95°C at a rate of 1°C per 3 seconds.
